# Two sides of the same algorithm: a qualitative study of ART professionals’ and patients’ views on the use of machine learning for embryo assessment and selection

**DOI:** 10.1007/s10815-026-03932-z

**Published:** 2026-06-17

**Authors:** Amy N.S. Webb, Andrea Whittaker, Julian J. Koplin, Cal Volks, Catherine Mills, Molly Johnston

**Affiliations:** 1 Monash Bioethics Centre, Monash University, Clayton, Australia; 2 School of Social Sciences, Monash University, Clayton, Australia

**Keywords:** Artificial intelligence, Embryo assessment, Machine learning, Attitudes, Trust

## Abstract

**Purpose:**

Machine learning (ML) is increasingly being introduced into assisted reproduction clinical practice, particularly for embryo grading and selection. This study aimed to explore how assisted reproductive technology (ART) professionals, regulators and patients understand the use of ML in embryo assessment.

**Methods:**

Semi-structured interviews were conducted with ten ART professionals/regulators and ten patients undertaking assisted reproduction in Australian clinics. Interviews explored themes identified as ethically salient to clinical implementation.

**Results:**

Both professionals and patients expressed mistrust of ML and emphasised the importance of human oversight in embryo selection. Participants identified uncertainties regarding how ML produces knowledge and raised questions about accountability and responsibility for ML-assisted decisions. Concerns were expressed that ML may lead to the discarding of viable embryos in the pursuit of selecting embryos. Participants called for full disclosure from clinics regarding the use of ML. Most professionals expressed a preference for transparent ML models and raised concerns about incorrect scoring due to poorly trained algorithms, handling errors, and potential algorithmic bias. Some professionals also expressed concern about over-reliance on ML and the potential for workforce deskilling.

**Conclusion:**

ART professionals and patients are cautious about the introduction of ML into embryo selection and emphasize the continued importance of human oversight, transparency, and accountability. Knowledge production with ML remains contested and there is uncertainty on how best to incorporate ML and reconfigure the work of embryologists and their professional expertise.

**Supplementary Information:**

The online version contains supplementary material available at https://doi.org/10.1007/s10815-026-03932-z.

## Introduction

In conventional assisted reproductive technology (ART), embryologists assess embryo quality using standardized grading systems such as the Gardner score. Despite standardized criteria, grading is inherently subjective, and prone to inter- and intra-observer variation [1]. Machine learning (ML), a type of artificial intelligence (AI), has been proposed to enhance the consistency and objectivity of embryo assessment. These systems work by analysing time-lapse or still images of developing embryos and making predictions on their potential to lead to a successful outcome. Some studies suggest ML can match or surpass embryologist performance [2, 3]. A recent clinical trial showed no significant difference in clinical pregnancy rates following ML versus manual assessment [4]; ML use was, however, associated with efficiency gains, which may contribute to its appeal in clinical practice [4].

Beyond clinical performance, ML for embryo assessment raises a set of interrelated ethical and practical challenges that are central to its clinical adoption. Many models are “opaque,” with decision-making processes that are not readily interpretable, creating uncertainty around how outcomes are generated and who is accountable for them [5]. These concerns intersect with broader issues, including the risk of algorithmic bias, the potential for over-reliance on automated systems, and questions of equitable access, particularly where such technologies are introduced as premium add-ons within an already stratified sector [6–10]. At the same time, integrating ML into routine practice is not straightforward, as models often require local validation and adaptation, alongside continued human oversight [11]. These challenges highlight that the successful implementation of ML in embryo assessment will depend not only on demonstrated clinical benefit, but also on addressing concerns around transparency, accountability, and equity.

Understanding how these issues are perceived by those directly involved in ART is critical to informing how ML is introduced and integrated into practice. However, very few studies have examined stakeholder perceptions of ML in embryo assessment. Palmer et al. [12] surveyed 702 clinical embryologists from across 90 countries and reported a general openness to adopting AI (73.4%), including embryo assessment, in the ART laboratory. This willingness, however, was tempered by uncertainty or scepticism over performance and just under half (46%) of respondents also expressed concerns about the ethical, legal, and social implications of AI (although these were not explored in detail). These findings suggest that, despite growing interest, there remains considerable uncertainty about the challenges posed by ML, and how these technologies should be evaluated, governed and integrated into clinical practice.

Australia provides a useful setting in which to examine these issues. It has high utilisation of assisted reproduction [13], ARTs are publicly subsidised, and several clinics are involved in the development and deployment of ML technologies for embryo assessment. In this study, we sought to gauge the reception of ART professionals, regulators and patients to this technology and its implementation into practice. Given the current hype around ML technologies [14] and the accelerated concerns around assisted reproduction generally [15], there is a need to better understand the social, ethical, and practical implications of ML adoption in ART, as experienced by key stakeholders. This study provides valuable insight into how innovations are being introduced and integrated into assisted reproduction clinical practices.

## Methods

This article reports findings from qualitative interviews conducted with ten patients and ten ART professionals/regulators. Ethics approval was obtained from Monash University, Human Research Ethics Committee (project ID: 37,647) on 23 March 2023.

### Participants

ART patients who have had or were currently receiving treatment that involves the creation of an embryo (i.e. IVF or ICSI) at an Australian clinic were eligible for an interview. ART professionals either practising in an Australian-based ART clinic currently using ML for embryo assessment, or working in a regulatory role, were eligible to participate. Participants were recruited between April 2023 and July 2023 through known networks, parenting forums and social media.

### Data collection and analysis

The semi-structured interview script covered topics identified as ethically salient to the clinical implementation of ML embryo assessment [6] (Supplementary File 1). Explanations of the ML models and their current use in Australia were provided as part of the interview and prior to their relative questions. Verbal consent was obtained at the start of the interview.

Interviews transcripts were uploaded to NVivo, and analysed using an inductive content analysis approach (ICA) [16]. ANSW read and coded all interviews by identifying “big picture” categories, then did a second round to refine the larger codes into detailed codes as per the ICA approach. MJ followed this same method on a subset of the interview transcripts to ensure coding consistency. ANSW, MJ and AW discussed categories to address any discrepancies in identified codes and to reach a consensus on the final major categories.

## Results

The sample of ART patients was aged between 28 and 51 years old. At the time of the interview, two patients had completed ART treatment, and eight were current patients. All patients resided and received/were currently receiving treatment in the state of Victoria. All ART professionals interviewed had 10 or more years of experience in the field of ART with varying states of practice and roles (Table 1).

**Table 1:** The location of practice and current occupation of ART Professionals (*n* = 10)

Location of practice	*n* (%)
Victoria	6 (60)
New South Wales	1 (10)
Australian Capital Territory	1 (10)
Queensland	2 (20)
Occupation/role	*n* (%)
Embryologist	2 (20)
Regulator*	2 (20)
Clinician	3 (30)
Lab Manager	2 (20)
Scientific Director*	2 (20)

*One participant held both a clinical and regulatory role in the Australian ART sector

In what follows, we present the main categories identified across both professional and patient interviews.

### Anthropomorphising ML

Across both cohorts, ML was often anthropomorphised within the narratives as a member of a “team” in the laboratory. When asked about integrating ML into embryo assessment, most patients were open to the idea, provided an embryologist oversees the ultimate decisions about which embryos were considered the best to transfer.

What I think about straight away is comparing the ability of the ML to make a decision compared to the embryologist, and obviously, like to me, I think you wouldn’t use the ML in isolation; it would have to be like a team approach between the ML and the embryologist. – Patient 3

The undefined “human element” of the embryologist was seen as a failsafe for what is perceived as an unreliable and unvalidated technology. Most ART professionals interviewed considered ML as a valuable support tool, emphasising the agentive aspect of decision-making it entails.

A lot of us approached it with a lot of hesitation or caution, but I think we are leaning towards using it as a support tool. For example, normally if we’re unsure we will call our colleague over and ask for a second opinion, whereas now it’s almost like that’s our backup reassurer – ART Professional 1

### Trust and distrust of ML technologies

Despite ML being viewed by professional staff as a valued “team member”, there was a reluctance among professionals to relinquish control to ML in embryo assessment. For professionals who had worked closely with ML, concerns arose from first-hand experiences, including challenges such as incorrect scoring, the influence of environmental factors such as the placement of embryos in the incubator on assessment, and potential biases within the algorithm. As one professional noted, “the scoring system is a bit flawed” (ART Professional 3), while another acknowledged, “ML is not always correct” (ART Professional 8). The scientific backing of ML embryo assessment was questioned:

One of the main things I’m conscious of with particularly the static-image ML is some of the data that’s presented, I don’t have a great deal of confidence in the way that data has been analysed for publication and presentation. So, I’m not convinced that the perception of how effective that technology is necessarily accurate. – ART Professional 9

Subsequently, professionals positioned the embryologist figures as a primary source of authority and trust rather than the non-human ML technology, and indicated a preference for integrating ML merely as a decision aid, given their concerns about its reliability.

I think, if ever, the emphasis was changed and ML was given precedence over the embryologist, I would have real concerns about it, because I don’t think it’s always accurate. I think – I would always wish to – I think it’s very important that we always believe the embryologist first. – ART Professional 8

Patients also expressed concern about the readiness of ML to be used without oversight. Patients felt that the technology was yet to be validated and needed extensive further research:

I think we have to have pretty good high-quality studies, comparing that ML to the gold standard, which is your embryologist… We know that it takes 17 years for medical research to get into clinical practice, so I would say that’s a few decades off, in my opinion.–Patient 3

For me it would just be quantity [of times used], so like, I think time–the time would be important for X number of years to pass, so for the ML algorithm to kind of grade enough embryos and–for all of that data, you know? Like pregnancy rates, miscarriage rates, all of that data to be correlated within the ML algorithm, so after a certain period of time, you know, after 10,000 embryo trans–grades and transfers, and the data from that, then I would feel confident in the ML.–Patient 8

### Accountability

The scepticism towards ML not only stems from concerns about published data but also from the potential medico-legal impact on accountability for misleading claims and the implications of errors arising from the use of ML in embryo assessment. Embryology involves high stakes for patients, a responsibility that embryologists keenly feel, highlighting the potential negative impact on patients if the technology is implemented incorrectly:

At best you are delaying the patient’s [treatment] - if the wrong embryo was transferred. But at worst if you end up discarding something or making the decision not to free something, you are actually severely affecting the patient’s outcome [ie of a successful pregnancy]. - ART Professional 1

Professionals largely agreed that clinics should be held accountable for any errors resulting from the use of ML in embryo assessment. They also believe that professionals using the technology should be responsible for verifying the ML’s decisions:

At the moment 100 per cent us [are accountable], because our – as far as I know unless something has happened really recently there is no TGA authorised device that is allowed to make any decisions for us. -ART Professional 9

Although several patients also suggested the clinic should be accountable as they were “responsible for that tool” (Patient 10), other patients did not feel that the clinic should be held accountable and rather felt that the responsibility for error lies in the ML itself, and the manufacturer of the technology should be held responsible.

You can’t hold the lab responsible, just like you really can’t hold the lab responsible now. Like, if they grade an embryo X, Y, Z, it’s sort of acknowledged universally that it’s subjective. You certainly can’t sue a lab, you know, for that. – Patient 8

Some professional staff suggested that accountability and fault can, in practice, never be accurately attributed given the multiple factors that affect embryo development and the subjective nature of embryo assessment:

I think that’s a really difficult one …trying to prove that the AI was at fault, as opposed to a human assessing the morphology would be extremely difficult to tell. – ART Professional 8

### Loss of human-ness

The majority of patients stated concern about how the use of ML will impact the “human-ness” of the ART patient experience. Patients often stated that human connection and empathy are critical during such an emotional medical and social experience. Embryos can be understood as carrying a special status as potential future children, adding to the emotional weight and sensitivity to the issue. Patients were concerned about how ML will rank embryos that may not look like the ideal model but may still have a chance to lead to live birth.

I guess if that is the case with embryos differing so significantly, are we relying on technology to be able to make all of the judgements that maybe an embryologist might be able to say although maybe none of these embryos look ideal, I have seen in the past an embryo that looks like this has been successful. Which I guess in aspects of where you’re talking the very minute population that have a bit of quirky embryos and things will then ML be able to detect that or will that not be programmed into it. – Patient 10

I’d want it, along with the embryologist’s final opinion, because I don’t think—I think human knowledge, of years, in the end, will just add that reassurance.—Patient 2

Some of the ART professionals shared these opinions and worried about the possibility of ML disregarding embryos that would otherwise have not been if treated under the embryologist’s discretion.

That’s what I worry about with a computer selecting embryos that it’s going to go, ‘Oh No!’ that one’s not going to be able to do anything. If our system was dominated by ML, it may stop those babies being born that would have, could have, should have been born – ART Professional 2

ART Professionals reiterated the importance of retaining “human-ness” and empathy during embryo selection. Such professionals emphasised the irreplaceable roles of the clinician and embryologist in providing care for patients:

But it’s not going to be able to tell a patient they’ve had a really shit result and show them their embryos and how it’s got to that point. I find that all – a little bit nefarious over-dramatisation to think that we’re not – we’re reducing our human element. Certainly, I can’t imagine a scenario – we can have protocols and practices and assistance from our technology, but nothing is going to replace the clinician and the embryologist talking to the patient about their results. – ART Professional 10

### Disclosure

Another shared theme across the cohort was the importance of disclosure of the use of ML for embryo assessment and the need for the disclosure of the storage of bioinformation and scoring. Some professionals suggested that the time of consent would be an appropriate time to discuss the use of ML and address any questions or concerns about the technology:

I mean, we’re very big on informed consent, full disclosure, and respect. So, there would be a question asked, ‘Do you need more information? Can we help you understand?’ – ART Professional 5

I think the more information that we can give a patient, without freaking them out, I think the better. Even just to give them the heads up that this is what we’re actually using and why we’re using it. I don’t see a problem with it. -ART Professional 2

Some patients felt that the technology’s limitations also should be made clear to patients during these discussions. Particularly, on what ML can and cannot do, any known biases associated with the algorithm, and how the ML’s scores will be used and stored:

Really at the end of the day if you’ve got a happy, healthy baby it doesn’t really matter, but if there was some bias towards it in the beginning then I think that people should be told. –Patient 7

The focus on data and data privacy was a theme particularly important to the patient cohort. In the context of ML for embryo assessment, patient data plays a crucial role in validating the technology for clinical use. All participants expressed a willingness to contribute their data if it would help improve the ML model and support other patients undergoing treatment but emphasised that data privacy should be carefully maintained alongside data aggregation:

I think we all should be contributing to research, and it’s not like it’s hard for us to do that. I know some people might have concerns about privacy, but I think it’s quite easy to protect people’s privacy in these situations – Patient 3

ART professionals explored two areas in greater depth compared to the patient cohort: their preference regarding model transparency and workforce implications.

### Opaque (black box) ML models vs transparent (interpretable) models

Opinions on opaque ML models among ART professionals were divided. Some appreciated the potential of these models to detect unknown factors, potentially leading to new insights. As one professional noted:

I actually think [opaque models] it’s a really good thing, because with the transparent version you’re essentially just getting someone to build on your own knowledge. Whereas with the black box, what I really want to know is what is it actually looking at. Is it looking at what we think should be happening, do we really want two-cell embryo to cleave at 20 hours, is that what we want? Or is it picking up something completely that we’ve never looked at ever, it’s just too bad we don’t know? – ART Professional 1

However, most preferred transparency to understand how the technology works and why an algorithm suggested a particular grading. The main advantage of opaque models is their ability to uncover unknown factors, but the drawbacks include the risk of errors and the challenge of validating ML decisions, raising concerns about accountability and reliability:

I think opaque—I don’t think it needs to be completely transparent, but a black box [model] isn’t going to work. You need to understand what it is visualising so that you can ensure the quality control, that it has got that level of visualisation that it needs. - ART professional 5

I am always after information. So, I would want to know why—how it’s making those decisions. If it’s an opaque system, it could be just making crap up. I’d have no idea, and I just don’t trust that. - ART professional 2

However, this preference might be influenced by their knowledge of the technology, with two participants pointing out that a truly transparent ML model may not actually exist.

You can’t see how it arrived at that decision because there are just so, so many datapoints being used so many times. So, yes, people talk about transparent ML, I don’t think it’s actually possible. - ART Professional 6

### Knowledge production and workforce implications

Several of the comments from the professionals highlighted contestation over knowledge production and interpretation and the practices adopted by embryologists to produce consensus decisions. In their responses, the professionals described how varied aspects such as the correct placement of petri dishes under the embryoscope for example affect how ML works in real conditions—or in other terms how knowledge is co-produced between human and ML systems:

So, if you didn’t understand that, that it was looking at all of the images over time and you didn’t understand that the quality of the dish setup would influence its outcome, then you could be led very astray by a score that is discordant with what you could visually see. - ART Professional 5

At some point it might make decisions based on the new input that it incorporates that are simply wrong. So, that’s the danger, in my opinion. So, if you have a model, you validate it, it works [right], you use it. But if that model continues to learn and progress then you’ve got to be very careful that it doesn’t pick something up that is actually completely wrong and the results that are totally invalid. - ART Professional 6

Some interviewees also expressed concerns about over-reliance on the technology and how it might affect an embryologist’s ability to make informed decisions and eventually lead to a de-skilled workforce:

I think newer embryologists that are coming through and into [the work] - are relying more heavily on the use of ML - ART Professional 2

In contrast, some ART professionals noted that the use of ML for embryo assessment can present as an opportunity for staff to deepen their knowledge of embryology, noting that junior staff members are more receptive to ML, because it aids in developing their expertise and reducing the pressure of individual accountability.

## Discussion

This study is the first to compare ART patient and professional views on the use of ML for embryo assessment. Across both groups, there was a notable convergence of views, particularly regarding mistrust of ML, the perceived need for continued human oversight, and the importance of disclosure and accountability. At the same time, some concerns were more prominent to each cohort, with patients emphasising the importance of data privacy, and professionals raising concerns around model transparency, and workforce implications. Taken together, our findings highlight that the integration of ML into ART is not simply a technical challenge, but a socially negotiated process shaped by how these technologies are understood, trusted and incorporated into clinical practice.

A central finding was a shared sense of mistrust in ML, consistent with broader concerns surrounding AI in healthcare [17]. In the context of embryo assessment, this distrust appears to be amplified by the high stakes and perceived irreversibility of decision-making. Participants expressed concern that errors in ML assessment could result in the discarding of embryos that may have led to a healthy child, underscoring the moral weight attached to these decisions. This highlights how trust in ML is not solely informed by clinical performance, but by the consequences of its potential failures. As noted by Shevtsova [18], trust in the application of ML in medical settings is influenced by factors such as perceived safeguards, uncertainty, familiarity with the technology and the impact on professional roles. Our findings suggest that concerns across each of these domains remain salient in ART.

In response to many of these concerns, participants emphasised the importance of maintaining the “human element” in embryology as a necessary safeguard against what is perceived as an unreliable and still-untrusted technology. Despite some participants attributing some forms of agency or decision-making authority to ML, there was a notable reluctance to relinquish full control over embryo selection, reflecting broader preferences for human-mediated deployment of AI in healthcare [19, 20]. This highlights a broader tension between different forms of objectivity. Participants appeared to place greater trust in forms of objectivity grounded in professional expertise and strict protocols, something Perotta [11] refers to as a form of “mechanical objectivity.” By contrast, “algorithmic objectivity” was viewed by participants with greater suspicion due to its opacity and limited interpretability. This aligns with broader arguments that interpretable AI should be prioritised in clinical contexts, as the ability to understand and interrogate outputs is central to maintaining trust and accountability [21]. However, our findings suggest that interpretability alone may not be sufficient, as participants privilege forms of judgement grounded in human expertise, even where these are recognised as imperfect. These findings reinforce the importance of maintaining human oversight in embryo assessment, and position ML as a support to, rather than replacement for, professional expertise.

Given the importance of preserving trust and human oversight, transparency and effective communication are critical to the successful implementation of ML. Both ART professionals and patients emphasised the need for clear disclosure when ML is used in embryo assessment. For patients, this extended to the use of their data in the development and application of ML systems—an issue that is especially salient in light of recent data breaches at Australian clinics [22]. ART patients place high importance on scientific evidence in making their decisions and rely on the supply of clear information regarding risks, benefits and clinical utility [23]. However, effective communication relies on the knowledge and confidence of ART providers, which has been shown to be variable [12]. Participants’ accounts suggest disclosure may not be straightforward; even where models are described as “transparent,” the underlying logic of ML-generated scores may remain difficult to interpret in practice, especially within the constraints of embryologists’ training and time. This, along with general uncertainty about how ML systems function, may limit providers’ ability to address patient concerns or explain the technology’s implications. These findings underscore the need for ongoing professional education and training, as well as consideration of how embryologists’ workloads are structured, to ensure they have sufficient time and capacity to interpret ML outputs and communicate their implications effectively. Ensuring patients are adequately informed and able to meaningfully engage with decisions about ML use in their treatment will be critical to maintaining trust in the ART sector.

Also important to maintaining trust is appropriate regulatory oversight and governance structures. While ML systems have received regulatory approval for use through the Therapeutic Goods Administration in Australia [24], participants’ concerns suggest there remains limited guidance regarding how these technologies should be implemented, validated, monitored and overseen in practice. Both professionals and patients questioned whether current evidence supporting ML performance is sufficient for routine clinical use, with some participants expressing concern about flawed scoring systems, the quality of validation studies, and the possibility of performance drift. Questions of accountability also remained unresolved, particularly regarding responsibility for errors arising from ML-assisted decision-making. Together, these findings suggest that beyond demonstrating technical performance, successful implementation of ML in ART will require clearer professional and regulatory guidance regarding validation standards, ongoing auditing and monitoring, accountability frameworks, and the maintenance of meaningful human oversight.

Alongside these governance considerations, participants also reflected on the implications of ML for the workforce. The impact and possible disruption of AI on the workforce have been raised in both healthcare [25, 26], and embryology specifically [12, 27]. Our results suggest that these impacts may be experienced less in terms of job displacement, but rather a configuration of knowledge production and how expertise is operationalised in practice. ML reshapes embryology work by introducing new responsibilities related to interpreting, validating and managing algorithmic outputs. While some participants expressed concerns about potential deskilling, others welcomed the possibility that ML could reduce variability in grading and support clinical decision-making, especially for more junior staff. In this context, positioning ML as an adjunct to, rather than a substitute for, clinical judgement may help maintain embryologists’ sense of professional autonomy and responsibility. At the same time, integrating ML into practice may disrupt established workflows and require additional labour, complicating claims that these technologies deliver efficiency gains. These findings suggest that the successful integration of ML is likely to depend on recognising and, if necessary, resourcing the evolving roles of involved professional staff.

This explorative study has several strengths. The semi-structured qualitative approach allowed for in-depth exploration across two key stakeholder groups. Including ART professionals with over a decade of experience in various roles in various clinics, as well as patients at different stages of their treatment, allowed for a comparative analysis of views, providing a more comprehensive understanding of the social and clinical implications of ML use. The study’s limitations derive from the relatively small sample size, consistent with its exploratory qualitative design. As such, these findings are not intended to represent consensus views across the ART sector. Additionally, most participants were from the state of Victoria and the healthcare professionals involved were all highly experienced. Perspectives may differ across jurisdictions, clinic settings, and levels of professional experience.

In conclusion, this study suggests that while there is interest in the potential benefits of ML for embryo assessment, both ART professionals and patients remain cautious about its use. Concerns regarding trust, transparency, accountability and the preservation of human expertise appear to be key issues for implementation. Addressing these concerns will be essential to ensure that the implementation of ML into ART is both clinically effective and socially acceptable.

## Supplementary Information

Below is the link to the electronic supplementary material.

ESM 1 (DOCX 21.5 KB)

## References

[1] Paternot G, Wetzels AM, Thonon F, Vansteenbrugge A, Willemen D, Devroe J, et al. Intra-and interobserver analysis in the morphological assessment of early stage embryos during an IVF procedure: a multicentre study. Reprod Biol Endocrinol. 2011;9(1):127. 21920032 10.1186/1477-7827-9-127 PMC3181205

[2] Khosravi P, Kazemi E, Zhan Q, Malmsten JE, Toschi M, Zisimopoulos P, et al. Deep learning enables robust assessment and selection of human blastocysts after in vitro fertilization. NPJ Digit Med. 2019;2(1):21. 31304368 10.1038/s41746-019-0096-y PMC6550169

[3] Uyar A, Bener A, Ciray HN. Predictive modeling of implantation outcome in an in vitro fertilization setting: an application of machine learning methods. Med Decis Making. 2015;35(6):714–25. 24842951 10.1177/0272989X14535984

[4] Illingworth PJ, Venetis C, Gardner DK, Nelson SM, Berntsen J, Larman MG, et al. Deep learning versus manual morphology-based embryo selection in IVF: a randomized, double-blind noninferiority trial. Nat Med. 2024;30(11):3114–20. 39122964 10.1038/s41591-024-03166-5 PMC11564097

[5] Räz T. ML interpretability: simple isn’t easy. Stud Hist Philos Sci. 2024;103:159–67. 38176132 10.1016/j.shpsa.2023.12.007

[6] Koplin JJ, Johnston M, Webb AN, Whittaker A, Mills C. Ethics of artificial intelligence in embryo assessment: mapping the terrain. Hum Reprod. 2025;40(2):179–85. 39657965 10.1093/humrep/deae264 PMC11788194

[7] Afnan MA, Liu Y, Conitzer V, Rudin C, Mishra A, Savulescu J, et al. Interpretable, not black-box, artificial intelligence should be used for embryo selection. Hum Reprod Open. 2021;2021(4):hoab040. 34938903 10.1093/hropen/hoab040 PMC8687137

[8] Van de Wiel L. The datafication of reproduction: time‐lapse embryo imaging and the commercialisation of IVF. Sociol Health Illn. 2019;41:193–209. 31599989 10.1111/1467-9566.12881 PMC6856688

[9] Geampana A, Perrotta M. Predicting success in the embryology lab: the use of algorithmic technologies in knowledge production. Sci Technol Human Values. 2023;48(1):212–33. 36504522 10.1177/01622439211057105 PMC9727110

[10] Homanen R, McBride N, Hudson N. Artificial intelligence and assisted reproductive technology: applying a reproductive justice lens. Eur J Womens Stud. 2024;31(2):262–76.

[11] Perrotta M. Seeing through machines: algorithms and the pursuit of mechanical objectivity in embryo imaging. In: Arnaldi S, Crabu S, Viteritti A, editors. Bodies and technoscience. Practices, imaginaries and materiality. Trieste: University Press Italiane; 2024. pp. 41–59.

[12] Palmer GA, Paredes O, Drakeley A, Chavez-Badiola A, Woolley TE, Kaouri K, et al. Use and understanding of AI in the ART laboratory: an international survey. Reprod Biomed Online. 2025;50(3):104435. 39939196 10.1016/j.rbmo.2024.104435

[13] Mazi M, Temple-Smith P, Mol B. Is the use of IVF in Australia appropriate? Hum Reprod. 2022;37(Suppl. 1):deac107.

[14] Cohen J, Silvestri G, Paredes O, Martin-Alcala HE, Chavez-Badiola A, Alikani M, et al. Artificial intelligence in assisted reproductive technology: separating the dream from reality. Reprod Biomed Online. 2025;50(4):104855. 40287195 10.1016/j.rbmo.2025.104855

[15] Wallace, E. Rapid review of assisted reproductive technology and in vitro fertilisation regulation and accreditation in Australia. Victorian Department of Health; 2025. Accessed April 21, 2026. https://www.health.gov.au/sites/default/files/2025-09/rapid-review-of-assisted-reproductive-technology-and-in-vitro-fertilisation-regulation-and-accreditation-in-australia.pdf

[16] Vears DF, Gillam L. Inductive content analysis: a guide for beginning qualitative researchers. Focus Health Prof Educ. 2022;23(1):111–27.

[17] Quinn TP, Senadeera M, Jacobs S, Coghlan S, Le V. Trust and medical AI: the challenges we face and the expertise needed to overcome them. J Am Med Inform Assoc. 2021;28(4):890–4. 33340404 10.1093/jamia/ocaa268 PMC7973477

[18] Shevtsova D, Ahmed A, Boot IW, Sanges C, Hudecek M, Jacobs JJ, et al. Trust in and acceptance of artificial intelligence applications in medicine: mixed methods study. JMIR Hum Factors. 2024;11(1):e47031. 38231544 10.2196/47031 PMC10831593

[19] Larson DB, Harvey H, Rubin DL, Irani N, Tse JR, Langlotz CP. Regulatory frameworks for development and evaluation of artificial intelligence–based diagnostic imaging algorithms: summary and recommendations. J Am Coll Radiol. 2021;18(3):413–24. 33096088 10.1016/j.jacr.2020.09.060 PMC7574690

[20] Lekadir K, Frangi AF, Porras AR, Glocker B, Cintas C, Langlotz CP, Weicken E, Asselbergs FW, Prior F, Collins GS, Kaissis G. FUTURE-AI: international consensus guideline for trustworthy and deployable artificial intelligence in healthcare. BMJ. 2025;388. 10.1136/bmj-2024-081554 PMC11795397 39909534

[21] Hatherley J, Sparrow R, Howard M. The virtues of interpretable medical AI. Camb Q Healthc Ethics. 2024;33(3):323–32. 36624634 10.1017/S0963180122000664

[22] Whitson R. Genea patients want IVF giant held to account as cybersecurity expert raises ongoing concerns. *ABC News* Novemeber 12, 2025. Accessed April 20, 2026. https://www.abc.net.au/news/2025-11-12/genea-ivf-data-breach-fallout-ongoing-cyber-concerns-raised/105984716

[23] Lensen S, Hammarberg K, Polyakov A, Wilkinson J, Whyte S, Peate M, et al. How common is add-on use and how do patients decide whether to use them? A national survey of IVF patients. Hum Reprod. 2021;36(7):1854–61. 33942073 10.1093/humrep/deab098

[24] Therapeutic Goods Administration. Australian Register of Therapeutic Goods: Embryoaid (ARTG Entry 483070). Australian Government Department of Health and Aged Care. 2025. https://www.ebs.tga.gov.au/servlet/xmlmillr6?dbid=ebs/PublicHTML/pdfStore.nsf&docid=483070&agid=%28PrintDetailsPublic%29&actionid=1. Accessed 28 May 2026.

[25] Obermeyer Z, Emanuel EJ. Predicting the future—big data, machine learning, and clinical medicine. N Engl J Med. 2016;375(13):1216. 27682033 10.1056/NEJMp1606181 PMC5070532

[26] Aquino YS, Rogers WA, Braunack-Mayer A, Frazer H, Win KT, Houssami N, et al. Utopia versus dystopia: professional perspectives on the impact of healthcare artificial intelligence on clinical roles and skills. Int J Med Inform. 2023;169:104903. 36343512 10.1016/j.ijmedinf.2022.104903

[27] Bori L, Meseguer M. Will the introduction of automated ART laboratory systems render the majority of embryologists redundant? Reprod Biomed Online. 2021;43(6):979–81. 34753681 10.1016/j.rbmo.2021.10.002

