# Supplementary material for: Two sides of the same algorithm: a qualitative study of ART professionals’ and patients’ views on the use of machine learning for embryo assessment and selection
Source: J Assist Reprod Genet. 2026 Jun 17;43(8):2391–8. doi: 10.1007/s10815-026-03932-z (PMC13598045; doi:10.1007/s10815-026-03932-z)
Supplement: Supplementary file 1 [file 10815_2026_3932_MOESM1_ESM.docx]

**Supplementary file 1 - Interview schedule**

**For ART Practitioners**

**Demographic questions**

Thank you for your consent, I am going to start recording now and I would like to start the interview by getting to know you:

1. Can you tell me a bit about what you do for work?
   a. Prompts: where in Australia do you work? How long have you been in this role/in the industry?

**Views on AI**

Now I would like to ask you a few questions about your thoughts/views on the use of AI for embryo selection.

1. Can you tell me what you know about the use of AI for embryo selection?
   a. If no:
      i. Machine learning can be used to help embryologists select the best embryo to transfer by analysing large sets of data on embryo quality and success rates. This involves training a machine learning model on a dataset that includes information such as the appearance of the embryo under a microscope, the developmental stage, and the outcome of previous transfers.

The machine learning model can then use this data to identify patterns and make predictions about which embryos are most likely to result in a successful pregnancy. It is hoped that embryologists can use the output of the machine learning model to make more informed decisions about which embryos to transfer.

2. In the clinic you currently work in, is this technology available (either trialling or available to patients)?
   a. If no:
      i. Would you be interested in using the technology?
      ii. Do you have any reservations about its use?
   b. If yes:
      i. How do you feel about the technology?
      ii. Do you find it helpful in your day to day tasks?
      iii. Do you trust the technology?
      iv. Do you have any reservations about its use?
      v. How is it priced?
3. What clinical issues do you see arising if the use of AI for embryo selection becomes common practice in your clinic?
   a. How do you feel about discussing the use of AI for embryo selection with your patients? (prompts: confident? Unsure? anxious?)
   b. One possible form of AI system would be ‘opaque’ – i.e, the reasoning/algorithm for embryo selection is not made explicit to you. Another possible form would be ‘transparent.’ In this case, the system would be able to explain its reasons for selecting one embryo over another.
      i. How would you feel about an ‘opaque’ AI system for embryo selection? Would you be willing to use it?
      ii. How would you feel about a ‘transparent’ AI system for embryo selection? Would you be willing to use it?
      iii. Would you trust an opaque AI to select the best embryo vs your own clinical judgement?
      iv. If the AI was to come to a different conclusion compared to you, whose opinion would you favour? Yours or the AI? Would you override an AI’s decision? How would you resolve this issue?
4. What are some ethical or policy concerns you have about using AI for embryo selection?
   a. Do you have any medico-legal concerns about this use of AI ?
      i. Some people worry that the use of AI will remove the ‘human element’ from ART care. What do you think about this?
5. If the use of AI for embryo selection was something that was to be implemented in your clinic, based on an assessment of which factors would you trust the AI to be most effective/trustworthy in making the best embryo selection for transfer?
   a. Morphology?
   b. Morphokinetics?
   c. Biomarkers?
6. What do you see as the future of this technology?
7. Do you have any final comments/thoughts you would like to share? Or is there something you feel we should know about this that we haven’t asked?

**Questions specifically for ART regulators**

1. In the literature, there is some concern about liability and responsibility. Who do you think liability will fall to if something unexpected happens when AI has been used to select an embryo for transfer?
2. How do you think the use of AI for embryo selection will impact law/regulation in Australia?
   a. Do you think introducing AI for embryo selection will impact how clinics are accredited/RTAC requirements?

**For ART Patients**

**Demographic questions**

Thank you for your consent, I am going to start recording now and I would like to start the interview by getting to know you:

1. Can I please ask for your age and which state you are currently living in?
2. Where are you at in your fertility treatment?
   a. How many cycles have you undergone?
      i. What has been the outcome of these cycles?
   b. Do you know the cause of your infertility? Can you tell me about what prompted you to seek fertility treatment??
3. Can you tell me why you chose the clinic you are receiving treatment at?
   a. What things did you weigh up?
4. (probe) Were you interested in any particular treatment/intervention/technology that was on offer at this particular clinic?

**AI for embryo selection questions**

Thank you. Now I would like to ask you some questions on your views of the use of AI in embryo selection.

*In a traditional treatment cycle, embryologists monitor the growth of embryos in the lab. Embryologists will then select the best embryos for transfer based on their quality and developmental stage. They evaluate the appearance of the embryos under a microscope. The goal is to choose the embryo with the highest chance of implanting and resulting in a successful pregnancy.

Currently, the use of AI to select the best embryos for transfer is being trialled here in Australia and there is potential for this to become common practice in the futur*e. What this means is that the decision of which embryo to transfer would be made by AI instead of an embryologist.

1. Can you tell me what you know about the use of AI for embryo selection?
   a. Probes: have you heard about it before? Where or by who? ?

*It has been proposed that in the future, AI could be used to help embryologists select the best embryo to transfer by analysing large sets of data on embryo quality and success rates. This would involve training an AI model on a dataset that includes information such as the appearance of the embryo under a microscope, the developmental stage, and the outcome of previous transfers. The AI model could then use this data to identify patterns and make predictions about which embryos are most likely to result in a successful pregnancy.*

2. Would you be interested in having AI select your embryos?
   a. What is important to you when thinking about this?
3. How would you feel about having AI select your embryos in place of an embryologist?
   a. Would you feel more trust in either an AI model or an embryologist making this decision?
4. If the use of AI to select embryos were to become standard practice, what would you like to know about the technology before using it?
   a. Who do you feel would be best to inform you about the use of AI? The clinician? Nurse? Embryologist? Or a counsellor?
5. There are different types of AI models. Some models allow us to see how they arrived at their decisions and predictions. These are often referred to as ‘transparent’ models. However, some other models are ‘opaque’, which means that they don’t reveal how they made its decisions – in which case, it can be challenging to understand why the model chose one embryo over another.
   a. How much would you want to know about how the AI made its decision?
   b. Would you be willing to use an ‘opaque’ model that had good clinical outcomes, but could not explain why it chose one embryo over another)?
   c. If the AI made a mistake when selecting the best embryo to transfer, do you think someone should be held accountable for the AI failing?
      i. Can you explain why?
6. In order for AI to learn, they need access to a lot of information. To inform the decision about which is the best embryo, the AI will need pictures/videos or other data about patients undergoing treatment, their embryos, and treatment outcomes (e.g. pregnancy & live birth rates). These data would only be used to train the AI model; they would not be shared with anyone else.
   a. Suppose your clinic wanted to contribute your data. How would you feel about your data being used in this way?
   b. Would you be willing to contribute your information and/or your embryo's information to inform AI?
7. If AI was to become an add-on/option at an extra cost, under what conditions, if any, would you consider paying for it?
8. Do you feel that AI would change your ART experience? Can you explain how?
   a. Some people worry that the use of AI will remove the ‘human element’ from ART care. What do you think about this?
9. One concern about the use of AI models in healthcare is that their decisions might be informed by factors that some many people don't think should inform these choices. One example of this might be if an AI embryo selection tool was more likely than an embryologist to select male than female embryos.
   a. As a patient, would the use of an AI process such as this align with your values?
   b. Are there any factors that you wouldn't want an AI embryo selection tool to take into account?
   c. How would you feel if you used such a tool, and discovered after the fact that it did take such factors into account?
10. Do you have any final comments/thoughts you would like to share?
